# Predictions and Observations for the Oceanic Lithosphere From *S*‐to‐*P* Receiver Functions and *SS* Precursors

**DOI:** 10.1029/2018GL077675

**Published:** 2018-06-12

**Authors:** Catherine A. Rychert, Nick Harmon

**Affiliations:** ^1^ Ocean and Earth Science University of Southampton Southampton UK

**Keywords:** ocean, scattered waves, thermal, lithosphere‐asthenosphere, seismology, melt

## Abstract

The ocean lithosphere is classically described by the thermal half‐space cooling (HSC) or the plate models, both characterized by a gradual transition to the asthenosphere beneath. Scattered waves find sharp seismic discontinuities beneath the oceans, possibly from the base of the plate. Active source studies suggest sharp discontinuities from a melt channel. We calculate synthetic *S*‐to‐*P* receiver functions and *SS* precursors for the HSC and plate models and also for channels. We find that the HSC and plate model velocity gradients are too gradual to create interpretable scattered waves from the base of the plate. Subtle phases are predicted to follow a similar trend as observations, flattening at older ages. Therefore, the seismic discontinuities are probably caused by a thermally controlled process that can also explain their amplitude, such as melting. Melt may coalesce in channels, although channels >10 km thick should be resolvable by scattered wave imaging.

## Key Points

- Weak scattered phases are predicted from half‐space and plate models, increasing in depth with age, and flattening to 50–80 km
- A match with observed scattered wave depths, but not amplitudes, suggests a thermally controlled process, e.g., melt defines the plate
- Teleseismic scattered phases should resolve an LAB melt channel imaged by active source studies, suggesting greater complexity

## 1 Introduction

**Figure 1:**
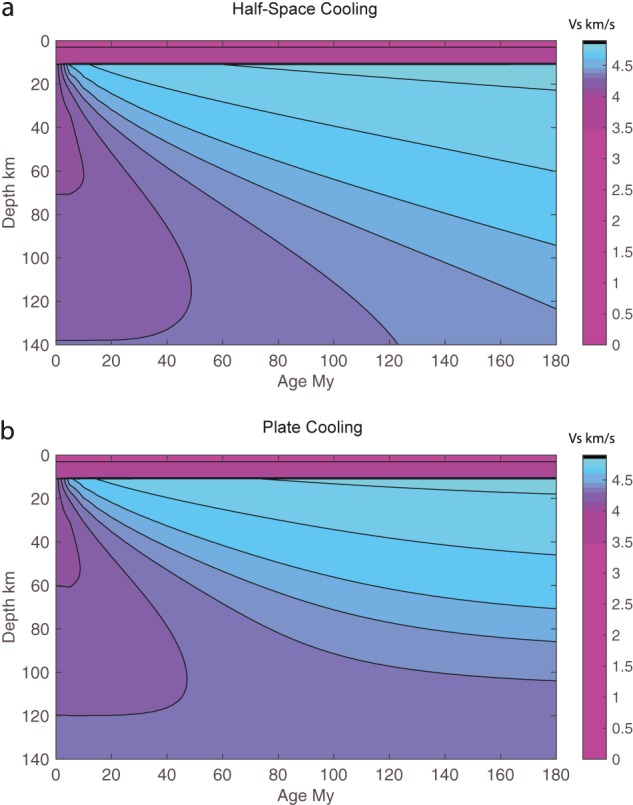
Predicted shear wave velocities. Predictions are calculated based on experimental relationships (Jackson & Faul, 2010) for the temperatures in the (a) half‐space cooling and (b) plate models. Grain size = 20 mm, potential temperature = 1,350 °C, and plate thickness = 100 km are assumed. There is a 3‐km‐thick water layer and a 7‐km‐thick uniform velocity crust.

Ocean plates cool, thicken, and subside with age, according to the square root of age relationship predicted by the half‐space cooling (HSC) model (Turcotte & Oxburgh, 1967), with more muted subsidence observed beneath the oldest seafloor, as in the plate model (Parsons & Sclater, 1977; Stein & Stein, 1992; Watts, 1978). Surface wave velocities increase with increasing seafloor age, as does the thickness of the seismically fast lithospheric layer in general agreement with HSC and plate models (Auer et al., 2015; French et al., 2013; Harmon et al., 2009; Nishimura & Forsyth, 1989; Priestley & McKenzie, 2013; Ritzwoller et al., 2004). The surface wave models are characterized by gradual velocity gradients from the lithosphere to the asthenosphere like those of the HSC and plate models (Jackson & Faul, 2010; Rychert et al., 2010; Tharimena, Rychert, Harmon, & White, 2017; Figure 1).

**Figure 2:**
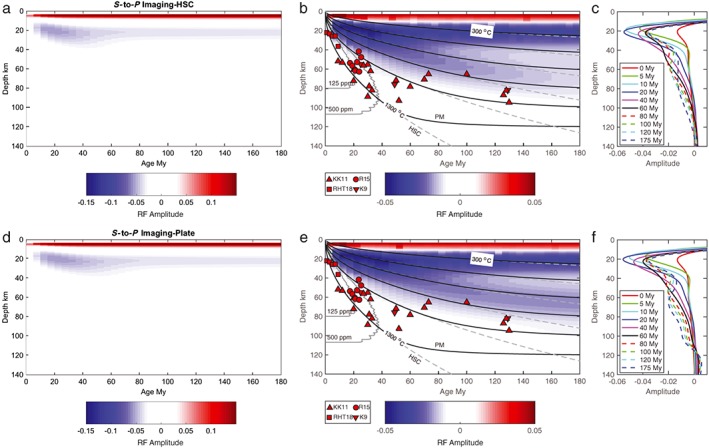
*S*‐to‐*P* receiver function predictions and observed lithosphere‐asthenosphere boundary depths. The red shading indicates a positive discontinuity or a velocity increase with depth, and the blue shading indicates a negative discontinuity or a velocity decrease with depth. Predictions for the half‐space cooling (HSC) model are shown (a) with a typical color scale, (b) with a saturated color scale to illuminate small amplitude arrivals, and (c) as waveform examples. Corresponding predictions for the plate model are shown in (d)–(f). The red symbols show *S*‐to‐*P* depths reported from previous studies, corrected from the sea surface to the seafloor by the amount indicated in parentheses as required KK11 (triangles; Kumar & Kawakatsu, 2011), K9 (inverted triangles; Kawakatsu et al., 2009), R15 (circles; −3 km; Reeves et al., 2015), and RHT18 (squares; −3 km; Rychert et al., 2018). The black lines show isotherms for the plate model. The gray dashed lines show isotherms for HSC. The gray lines near the ridge show the solidi for a mildly hydrated mantle, 125 and 500‐ppm water (Katz et al., 2003).

**Figure 3:**
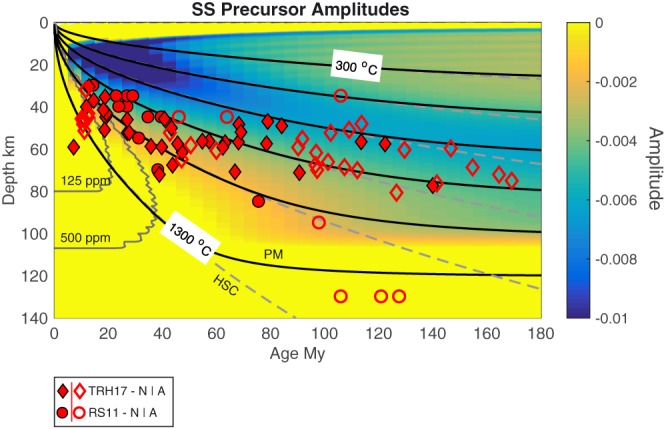
*SS* precursor predictions and observed lithosphere‐asthenosphere boundary depths. The background shading shows predicted *SS* precursor amplitudes of reflections from the plate model based on experiments (Jackson & Faul, 2010). The red symbols show depths of reported discontinuities from *SS* precursors TRH17 (diamonds; Tharimena, Rychert, Harmon, & White, 2017) and RS11 (circles; Rychert & Shearer, 2011) from anomalous (open) and normal (filled) seafloor after (Korenaga & Korenaga, 2008). The black lines show isotherms for the plate model. The gray dashed lines show isotherms for half‐space cooling. The gray lines near the ridge show the solidi for a mildly hydrated mantle, 125 and 500‐ppm water (Katz et al., 2003).

In contrast, several reports from reflected and converted body waves suggest strong, sharp negative velocity discontinuities, velocity decreases with depth (>5% *S* wave velocity drops over short depth ranges, typically <30 km), beneath the oceans often interpreted as the lithosphere‐asthenosphere boundary (LAB). Taken together *S*‐to‐*P* receiver functions and *SS* precursors image increasing discontinuity depths beneath young seafloor (Figures 2 and 3; Kawakatsu et al., 2009; Kumar & Kawakatsu, 2011; Reeves et al., 2015; Rychert & Shearer, 2011; Rychert et al., 2018; Schmerr, 2012; Tharimena, Rychert, Harmon, & White, 2017). Beneath older seafloor *SS* precursors, *S*‐to‐*P* and multiple *S* wave bounces are more consistent with a discontinuity at constant depth, ~60 km, although with some degree of scatter, at older ages (Gaherty et al., 1996; Kawakatsu et al., 2009; Schmerr, 2012; Tan & Helmberger, 2007; Tharimena, Rychert, Harmon, & White, 2017). There are also many reports from ocean islands, although these regions may not be representative of unaltered ocean lithosphere (Byrnes et al., 2015; Li et al., 2004; Lodge & Helffrich, 2006; Rychert et al., 2013, 2014; Rychert & Shearer, 2009; Vinnik et al., 2012). Active source studies have been used to argue for an even sharper discontinuity (8% *P* wave velocity drops over <1 km) at 72–88 km depth beneath the seafloor, accompanied by a deeper velocity increase of similar magnitude, interpreted as a 10 to 18‐km‐thick melt rich channel representing the base of the plate (Mehouachi & Singh, 2018; Stern et al., 2015).

These discontinuity observations from body waves and active source are not necessarily inconsistent with the surface wave models. The discontinuity depths generally fall within the gradual drops from surface waves, and surface waves cannot distinguish the difference between a gradual, ~40 km wide, gradient and a sharp velocity gradient. However, sharp velocity gradients are also not well‐predicted by either HSC or the plate model. In addition, exactly how channel features may be reconciled with numerous observations of singular negative velocity decreases is not known. Therefore, the exact relationship of the observations to the tectonic plate and also the implications for the definition of the plate are debated.

Here we translate HSC and plate models to seismic velocity using relationships from experiments (Jackson & Faul, 2010). We translate these to predictions for *S*‐to‐*P* receiver functions and *SS* precursors and compare the results to observations. Finally, we test the resolution of receiver functions and *SS* precursors to the channels that have been proposed at LAB depths by active source studies.

## 2 Methods

We calculated a series of predictions for the plate model and the HSC model to investigate the relationship of the aforementioned seismic observations to the tectonic plate. We constructed an HSC model and also a plate model assuming potential temperature 1,350 °C and plate thickness 100 km. We then translate the temperature model to seismic velocity, *Vp* and *Vs*, assuming experimental relationships (Jackson & Faul, 2010). We assumed a grain size of 20 mm, which is likely appropriate for the LAB depths beneath the oceans (Behn et al., 2009). We assumed a 3‐km‐deep ocean with *Vp* = 1.5 km/s, density of 1,000 kg/m^3^, a 7‐km‐thick crust with *Vp* = 6.5 km/s, *Vs* = 3.6 km/s, and density = 2,800 kg/m^3^ and a mantle density of 3,300 kg/m^3^. The mantle was discretized every 2 km in depth in the upper 400 km.

We calculated synthetic seismograms for *S*‐to‐*P* phases (Shearer & Orcutt, 1987) and *SS* precursors (Keith & Crampin, 1977) through the seismic velocity models predicted by experiments for HSC and the plate model. We assumed typical slowness values, 0.095 s/km for *S*‐to‐*P* and 0.11 s/km for *SS*. We calculated seismograms for ocean bottom stations located on the seafloor for receiver functions and for reflections for *SS* for seafloor ages 0 to 180 Myr at 5‐Myr increments.

For *S*‐to‐*P* we used a typical high quality *S* wave example recorded at an ocean bottom station during the Cascadia Initiative (Toomey et al., 2014) and previously used to image the base of the plate (Rychert et al., 2018). We found that any high‐quality *S* wave gave the same answer given the filtering and deconvolution parameters applied. We then deconvolved the receiver functions using a simultaneous deconvolution and a typical band‐pass filter (0.02–0.25 Hz) and migrated the receiver functions to depth in 1‐D assuming the input velocity model (Bostock, 1998; Rychert et al., 2005, 2007). We fliped polarity of the receiver functions to be consistent with *P*‐to‐*S* imaging, where a positive phase corresponds to a velocity increase with depth and a negative phase corresponds to a velocity decrease with depth. The *S*‐to‐*P* receiver functions are presented using a color scale we would typically use to present similar data and also a scale bar with reduced limits to emphasize small amplitude phases. Synthetic receiver function depths with respect to the seafloor and observed depths have been corrected for a water layer if necessary (see figure caption 2).

For *SS* we experimented with using the ocean reference waveform (Rychert & Shearer, 2010, 2011; Tharimena, Rychert, & Harmon, 2017; Tharimena, Rychert, Harmon, & White, 2017). However, because its sidelobes are so broad and the reflections from the HSC and plate models are so weak and broad, the LAB discontinuity reflections were indiscernible. To illustrate where these phases are expected to arrive and their apparent magnitude, we present a model with an unrealistic source waveform consisting of a simple Gaussian peak with a 15‐second period and no sidelobes, emphasizing that given this assumption, the phases we analyze in the synthetics might not be resolvable in real data.

We calculated synthetic seismograms at a low velocity channel discontinuity, that is, a negative polarity discontinuity (velocity decrease with depth) underlain by a positive polarity discontinuity. Although channel features are typically detected as *P* wave discontinuities, we assume that they are associated with a similar magnitude *S* wave contrast. This is likely the case in the absence of any knowledge of *Vp*/*Vs* ratios. Also given that *Vp*/*Vs* is expected to be high (not low) in a melt‐rich channel, *S* waves should be affected in the same way or more than *P* waves. We included a 7‐km‐thick crust (*Vp* = 6.5 and *Vp*/*Vs* = 1.75), a mantle lithosphere (*Vp* = 8.2 and *Vp*/*Vs* = 1.8), and an 8% velocity drop at the top of the channel at 72‐km depth. We used a velocity increase of 8% at its base. We tested a range of thicknesses by changing the depth of the base of the channel.

We did this for *S*‐to‐*P* waves assuming horizontal slowness 0.095 s/km, using a source from the Cascadia Initiative as described above (Toomey et al., 2014). We deconvolved the waveforms using an extended time multitaper method and using a broad range of low‐pass filters (0.1 to 2 Hz; Helffrich, 2006; Rychert et al., 2012), values that encompass those typically implemented by ocean bottom *S*‐to‐*P* receiver function studies, for example, 0.12 Hz (Reeves et al., 2015), 0.14 Hz (Rychert et al., 2013, 2014), or 0.25 Hz (Rychert et al., 2018). We used the multitaper parameters suggested by Shibutani et al. (2008).

For *SS* precursors we used the global ocean reference *SS* waveform (Rychert & Shearer, 2010, 2011; Tharimena, Rychert, Harmon, & White, 2017), assumed a slowness of 0.11 s/km, and tested a range of separation distances. We did not vary the low‐pass filter since we do not apply additional filtering in our *SS* methodology to minimize potential sidelobe artifacts (Rychert & Shearer, 2010).

**Figure 4:**
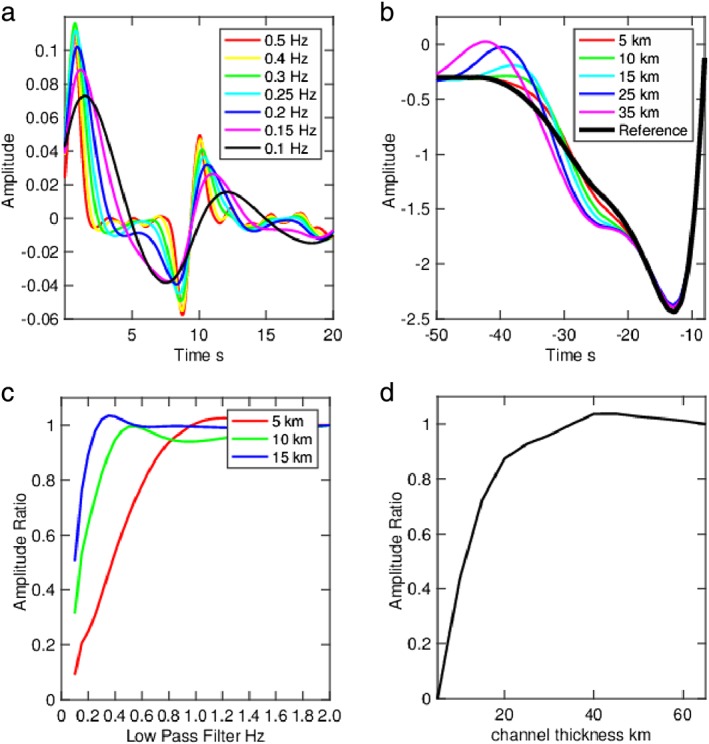
Channel resolution tests. (a) *S*‐to‐*P* receiver function waveform examples from a 10‐km‐thick channel assuming a range of low‐pass filters. (b) *SS* precursor waveform examples for a range of channel thicknesses (colored lines). The panel is zoomed in on the sidelobe of the main *SS* phase, and the main *SS* pulse is out of the frame to the right‐hand side. Reference *SS* waveform stack with no channel is in black. (c) Amplitude ratio of the *S*‐to‐*P* conversions for the base of a channel that is 5 km (red), 10 km (green), and 15 km (blue) thick for a variety of low‐pass filters in comparison to the amplitude for low‐pass filter = 2.0 Hz. (d) Amplitude ratio of the *SS* precursor from the top of the channel in comparison to the amplitude for a thickness where amplitude is completely recovered, 35 km or greater.

We compared the amplitude of the converted phases to the amplitude of a reference in which the phases from the top and bottom of the channel do not interfere. The reference phase for receiver functions is the positive conversion from the base of the channel filtered at the highest frequency (2 Hz). For each channel thickness, we compared the filtered receiver function amplitudes of the positive phase to the amplitude of its high‐frequency realization. The reference phase for *SS* precursors is the reflection from the top of a thick channel, >35 km. In both cases the amplitudes of the phases from the top of the channel are typically similar to those from the base (Figure 4). However, we chose the phase from the channel base for receiver functions to avoid interference with the Moho phase. We chose the phase from the top of the channel for *SS* precursors to avoid a more complicated interference pattern with the *SS* sidelobe.

## 3 Results

The predicted velocity models for HSC and plate models are similar (Figure 1). Both are characterized by increasing velocity and increasing thickness with age. The only difference is the shape of the velocity contours beneath the oldest lithosphere. HSC is characterized by fast velocities that extend to greater depths in comparison to the plate model.

The predicted receiver functions for HSC and plate models are generally characterized by low‐amplitude LAB signals (Figure 2). Beneath the youngest lithosphere (0–5 Myr), the LAB signal is weak since the plate is still relatively hot and seismically slow, and therefore, the magnitude of the velocity drop with depth is small. At intermediate ages (10–40 Myr) an *S*‐to‐*P* conversion interferes with the Moho sidelobe at 17 to 23 km depth, increasing its amplitude to (0.05–0.06), which could be misinterpreted as a 7–8% velocity contrast. However, the fact that it is indistinct from the sidelobe would make interpretation as an LAB phase tenuous. At older ages (>60 Myr) the phase near 20 km is diminished in amplitude because it is just the sidelobe of the Moho. The thicker lithosphere with a more gradational base produces low‐amplitude conversions. These arrive at 50–65 km depth for lithosphere ages ≥70 Myr in the plate model. The amplitudes of the conversions are consistent with <3% contrasts, that is, if they were caused by a single step function, rather than a gradient. These arrive at 50–80 km depth for lithosphere ages ≥70 Myr in the HSC model. The amplitudes of the HSC conversions are consistent with ~3% contrasts at 70 Myr, gradually decreasing to ~2% at 180 Myr.

The predicted *SS* precursors for the HSC and plate models are also small. Here we show the plate model example (Figure 3). A phase from 18–32 km depth arrives beneath ~10–40 Myr, a similar feature to that in *S*‐to‐*P* receiver functions, in the region where the plate has cooled, and is seismically fast, but the velocity gradient is relatively sharp in comparison to older ages. At older ages the phase increases in depth and then flattens to 50–65 km beneath >80‐Myr‐old seafloor. We emphasize that we can only see this phase owing to our use of a sidelobe‐free single pulse *S* wave, which is not realistic for normal data. If a more realistic source with wide sidelobes, such as the ocean reference stack is used, no clear LAB phase is observed owing to complicated interference with the sidelobe region of the *SS* phase. For this reason, we limit our presentation of *SS* precursor models and interpretation.

We find that low‐velocity channels that are 10 km thick or greater are predicted to be well‐imaged by *S*‐to‐*P* receiver functions. The predicted amplitude of the converted or reflected phases from the top and the bottom of the channel are completely or nearly completely recovered; that is, there is little to no destructive interference (Figure 4). This is true for typical low‐pass filters, that is, 0.25 Hz (Rychert et al., 2018) or 0.14 Hz (Rychert et al., 2013, 2014), and the phases may still be visible even in extreme cases as low as 0.12 Hz (Reeves et al., 2015) or even 0.1 Hz (Figure 4).

We find that *SS* waves would also resolve channels >10 km. The amplitudes of the phases for 15‐km‐thick channel would easily be interpretable and are also nearly the size of phases from a fully resolved 35‐km‐thick channel (Figure 4).

## 4 Discussion

No *S*‐to‐*P* phases from the LAB are predicted to be interpretable for either HSC or plate models given the small amplitudes of the predicted conversions. In one region where a large amplitude negative phase is predicted at 17 to 23 km depth for 10 to 40‐Myr‐old lithosphere, it is due to interference with the sidelobe of the Moho. Typically, interpretation of sidelobes such as this is avoided. For instance, beneath Cascadia, we found a LAB phase corresponding to a 5% velocity drop at 20 to 45 km depth beneath 0 to 10‐Myr‐old lithosphere (Rychert et al., 2018). However, we were able to rule out the possibility of a purely thermal model for two main reasons: (1) The Moho was artificially shallow because of interference with sediment conversions, which reduced the potential for interference with the LAB phase, and (2) the LAB phase clearly pulled away from the Moho and its sidelobe.

The predicted *S*‐to‐*P* receiver functions for the HSC and plate models are similar to each other. The weak amplitude LAB conversions roughly follow the 800 °C isotherm. The similarity occurs because the sharpest part of the predicted seismic velocity gradient occurs at a similar depth for both models, and this is what is reflected in the receiver functions. More gradual gradients at >80 km do not create converted phases that can be seen even in the case of the enhanced color bar (Figure 2). The depth trend of the small amplitude *S*‐to‐*P* phases increasing from 20 to 65 km (plate) or 80 km (HSC) depth resembles those from observations (Figure 2). The small‐amplitude phases are either in agreement or just shallower than the observations (Figure 2). This suggests that, although the thermal HSC model and the plate model cannot explain *S*‐to‐*P* observations of a sharp velocity gradient, a thermally defined model likely contributes in part to observed converted phases.

No *SS* precursors are predicted at interpretable levels for HSC or plate models. Small amplitude phases related to the negative velocity gradient are visible in the case where an artificial *SS* phase with no sidelobe is used. These phases arrive at similar depths, or just shallower than those reported for *SS* precursor observations (Figure 3). Again, this suggests that the HSC and plate models are insufficient to explain the amplitudes of the observations. However, the thermal model likely contributes, and temperature may control the process that does explain the velocity gradients required by the observations. Given that the *SS* precursor phases are not even discernible with realistic source phases, we have presented only one example case (plate model) and limit the length and depth of our discussion on them.

The exact amplitude of the predicted conversions and reflections depends to some degree on the assumptions. Here we used the parameterization of Jackson and Faul (2010), which is based on pure olivine experiments. Other modeling attempts that included a wide range of attenuation assumptions, the effects of dehydration, and a peridotitic bulk chemistry still found similar velocity profile characters (Goes et al., 2012). The total expected velocity drops at the base of the plate were like those presented here and lower (Goes et al., 2012), suggesting that the conversions from HSC or plate models may be even less than our predicted 2–3% apparent contrasts. Recently, a very high *Q*_*s*_ (3,000) was reported for western Pacific lithosphere at 1–3 Hz (Takeuchi et al., 2017), which when compared to surface waves (Dalton et al., 2008) suggests frequency‐dependent lithospheric attenuation. We did a test in which we imposed a lithospheric *Q*_*s*_ of 1,000 shallower than at the 900° isotherm in the *S*‐to‐*P* receiver function model. This is a conservatively high *Q*_*s*_ for the dominant periods of our seismic waves (~10 s) assuming the frequency dependence suggested by Takeuchi et al. (2017). We assumed attenuation from Jackson and Faul (2010) beneath. This caused a subtle increase (<0.015) in the amplitude of the receiver function conversion. The increase was strongest in the region where the LAB phase interferes with the sidelobe of the Moho at 10–35 Myr, corresponding to an additional contrast of 1.7%, although it was typically <1% in other areas. Finally, beneath intermediate and slow spreading ridges lateral heat conduction could create a thick lithosphere (Parmentier & Morgan, 1990; Rychert et al., 2018), which we have not accounted for here, although the main impact would only be an increase in the LAB signal beneath the ridge.

One possible explanation for agreement with the age‐depth trend and under prediction of amplitudes of the scattered wave observations is a thermal process controls the depth, with an additional factor such as partial melting of the asthenosphere. At young ages, <40 Myr, *S*‐to‐*P* and *SS* precursor observations roughly follow the hydrated solidus, roughly defined by the 1,100 °C isotherm. At greater ages, where no melt is predicted, it remains open. But the relatively flat trends in the observations and the predictions even for a purely thermal model suggest a thermally controlled underlying process. A subsolidus mechanism such as elastically accommodated grain boundary sliding (Karato, 2015) or anisotropy has been proposed (Auer et al., 2015; Beghein et al., 2014). However, whether or not grain boundary sliding can explain the seismic observations is debated (Cline et al., 2018; Jackson & Faul, 2010). In addition, anisotropy cannot explain large negative *S*‐to‐*P* conversions (Rychert & Harmon, 2017). Alternatively, melt may pond at a depth where it is neutrally buoyant, a process that would have a thermal control like our predictions (Sakamaki et al., 2013). Although temperatures at 50–80 km depth beneath >80‐Myr‐old seafloor are likely below the silicate melt solidus, they may exceed the carbonatitic solidus (Hirschmann, 2010; Tharimena, Rychert, Harmon, & White, 2017).

Melt may coalesce in channels as has been suggested by active source studies. However, a singular LAB melt channel is not easily reconciled with the observations from converted and reflected phases. We predict that both receiver functions and *SS* precursors would detect both edges of the channel. Whereas, typically, only negative signals corresponding to velocity decreases with depth are observed in receiver functions and *SS*‐precursors. It has been argued that receiver functions and *SS* waveform results could be complicated by a hydration induced grain boundary sliding effect, whereas the *P* wave reflections are seeing the true LAB melt channels (Karato, 2015; Mehouachi & Singh, 2018), although recent experiments suggest that seismic velocity is not affected by hydration, but rather the redox state of the mantle (Cline et al., 2018). In addition, exactly how the grain boundary sliding effect would perfectly mask the melt channels from receiver functions and *SS* precursors is not clear. An alternative explanation is that channels are not ubiquitous or that they are transient. Another possibility is that there is a more complex but consistent velocity structure in depth which we have not tested, perhaps involving multiple melt channels in depth.

## 5 Conclusions

Predicted seismic velocities for the HSC and plate models are similar and characterized by gradual velocity gradients at the base of the plate. *S*‐to‐*P* receiver functions and *SS* precursor phases converted and reflected from the gradual velocity gradient at the base of the plate in the HSC and plate models are similar. The predicted converted and reflected phases are too small for interpretation. However, the predicted depths of the small amplitude conversions and reflections are in good agreement with those from observations. This suggests that the observed depths are probably dictated by thermal process even if another mechanism is required to explain the magnitude of the observed velocity contrasts. For example, the observations are in good agreement with the damp peridotite solidus, at <40‐Myr seafloor, and could be influenced by melt. The melt may coalesce in a channel, although both the top and the bottom of low‐velocity channels are predicted to be well‐resolved by *S*‐to‐*P* receiver functions and *SS*. Reconciling channel observations with the consistent negative features in *SS* and *S*‐to‐*P* is not just an issue of resolution and suggests greater complexity or a yet to be discovered phenomenon.
